# *Gigantochloa glabrata* (Poaceae, Bambusoideae), a new bamboo species from Yunnan, China

**DOI:** 10.3897/phytokeys.171.59562

**Published:** 2021-01-07

**Authors:** Zu-Chang Xu, Jing-Xia Liu, De-Zhu Li

**Affiliations:** 1 Germplasm Bank of Wild Species, Kunming Institute of Botany, Chinese Academy of Sciences, Kunming, Yunnan 650201, China Kunming Institute of Botany Kunming China; 2 Kunming College of Life Science, University of the Chinese Academy of Sciences, Kunming, Yunnan 650201, China University of the Chinese Academy of Sciences Kunming China

**Keywords:** *
Gigantochloa
*, new species, paleotropical woody bamboos

## Abstract

*Gigantochloa glabrata* N. H. Xia & Y. Zeng ex D. Z. Li & Z. C. Xu, **sp. nov.**, a new species of paleotropical woody bamboo has been described and illustrated from Yunnan, China. The new species is morphologically similar to *G. albociliata* and *G. levis*, but differs from them by having erect culm sheath blades; culm sheath ligules 4–6 mm high, truncate, denticulate; and with a ring of white tomentum on the intranode and below the node. The new species was mistakenly identified as *Gigantochloa albociliata* in the *Flora of China* and was recognised with description of the vegetative characters in 2014, but it was not effectively published. Here, we designate a complete specimen with inflorescence as the type and describe it in accordance with the Code.

## Introduction

*Gigantochloa* Kurz ex Munro was published as a new genus by Kurz (1864) without any detailed description. Munro described the morphological characters of this genus and validated the publication (Munro 1868). Currently, there are more than 60 species recognised in *Gigantochloa* from all over the world, which are distributed in the tropical lowlands of Southeast Asia (Holttum 1958; Widjaja 1987; Vorontsova et al. 2016), with seven species recorded in China (Li et al. 2006; Zeng et al. 2014). Species of *Gigantochloa* are characterised by their pseudospikelets clustered at each flowering branch node, oblong or linear, each with 2–4 florets and one terminal imperfect floret consisting only of an empty lemma, rhachilla internodes obscure, paleas 2-keeled, the keels and the inflexed margins long-ciliate above; lodicules often absent; stamens six, the filaments connated into a hyaline tube which can elongate and become membranous, with anthers apiculate with minutely hispidulous tips (Munro 1868). Since its eastablishment, many bamboo taxonomists have considered it as a “good genus”, based on morphological characteristics (Kurz 1875, 1876; Holttum 1958; Clayton and Renvoize 1986; Widjaja 1987).

As a genus of paleotropical woody bamboo, *Gigantochloa* belongs to the subtribe Bambusinae Presl (BPG 2012). It was included in the *Bambusa*-*Dendrocalamus*-*Gigantochloa* (BDG) complex, together with *Bambusa* Schreber, *Dendrocalamus* Nees and closely-related small genera (Goh et al. 2010; Goh et al. 2013; Zhou et al. 2017). Morphologically, *Bambusa* can be distinguished from *Gigantochloa* by its conspicuous auricles and florets falling separately. *Dendrocalamus* can be recognised by its free filaments. In our recent molecular phylogenetic study, *Gigantochloa* was well resolved as a monophyletic group (Liu et al. 2020).

By studying the species of *Gigantochloa* from the Yunnan-Myanmar-Thailand floristic region, we found that *G. albociliata*, recorded in *Flora Reipublicae Popularis Sinicae* (Keng and Wang 1996) and *Flora of China* (Li et al. 2006) is not truly *G. albociliata* (Munro) Kurz. Accordingly, a new species needs to be described to clarify this long-existing taxonomic problem.

## Materials and methods

All measurements and observation of morphological characters were conducted, based on the specimens at the Herbarium of the Kunming Institute of Botany (**KUN**), Herbarium of the Xishuangbanna Tropical Botanical Garden (**HITBC**) and the Herbarium of the South China Botanical Garden (**IBSC**), as well as the photos of living individuals taken from living collections of the Xishuangbanna Tropical Botanical Garden in the summer of 2019. Pseudospikelets were dissected under an OLYMPUS DP80 digital microscope at Germplasm Bank of Wild Species of the Kunming Institute of Botany. Morphological comparisons with closely-related species (*G. albociliata* and *G. levis* (Blanco) Merr. (Blanco 1837; Merrill 1916)) were based on characters recorded in literature and on the type specimens. The morphological terminology follows McClure (McClure 1966).

## Taxonomy

*Gigantochloa albociliata* (Munro) Kurz was first recorded in Yunnan, southwest China by Sun (1984) and it was included in *Flora Reipublicae Popularis Sinicae* (Keng and Wang 1996), *Flora of China* (Li et al. 2006) and the *Flora of China Illustrations* (Zhang 2007). However, the description and illustrations of the *Flora of China* and the protologues of *G. albociliata* did not match. When we checked the specimens of *Gigantochloa* at the HITBC in 2019, we noticed that the inflorescence specimen, collected by *K. H. He* (no. *C130051*) in 2007, was identical with “*G. albociliata*” in the sense of *Flora of China*. We collected inflorescence material in the living collection of the Xishuangbanna Tropical Botanical Garden again in August 2019. After comparison with specimens of *G. albociliata* and other closely-related *Gigantochloa* species, we could not place it within any described species of *Gigantochloa*. In the meantime, we noticed that this species was recognised by Zeng (2014) as a new species with description of the vegetative characters. Zeng’s new name is available via the International Plant Names Index (IPNI 2020); however, according to the Code (Turland et al. 2018), it was not effectively published, because it appeared only in a thesis submitted to a university for the purpose of obtaining a degree, with neither an ISBN number nor statement of the name of the printer, publisher or distributor in the original printed version (Art. 30.9). Here, we added reproductive characters and a detailed morphological comparison to validate the new species as *G. glabrata* N. H. Xia & Y. Zeng. We designate a complete specimen with an inflorescence as the type and describe it in accordance with the Code.

After checking the type specimens and protologue of *Gigantochloa albociliata*, it is confirmed that the true *G. albociliata* is naturally distributed in southern Yunnan, China, as well as northern Myanmar and northern Thailand. In Yunnan, it often grows in mixed forest or roadside.

### Gigantochloa glabrata

Taxon classificationPlantaePoalesPoaceae

N. H. Xia & Y. Zeng ex D. Z. Li & Z. C. Xu sp. nov.

AD21867F-3319-5BE0-B5FA-37E4227A27F7

urn:lsid:ipni.org:names:77213497-1

**Figure 1.**
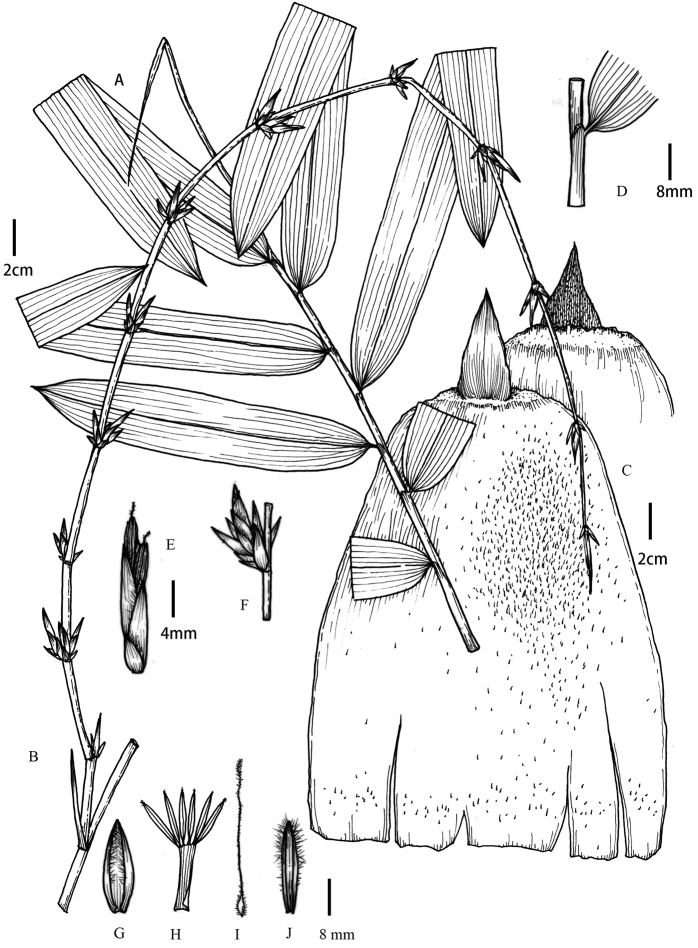
*Gigantochloa glabrata* N. H. Xia & Y. Zeng ex D. Z. Li & Z. C. Xu **A** leaf branch **B** flowering branch **C** culm sheath **D** leaf ligule **E** pseudospikelet **F** inflorescence **G** lemma **H** anthers **I** pistil **J** palea. Drawn from the type specimen and pictures by Yi-Fan Li. Scale bars: 2 cm (**A–C**); 8 mm (**G–J**); 4 mm (**D–F**).

**Figure 2.**
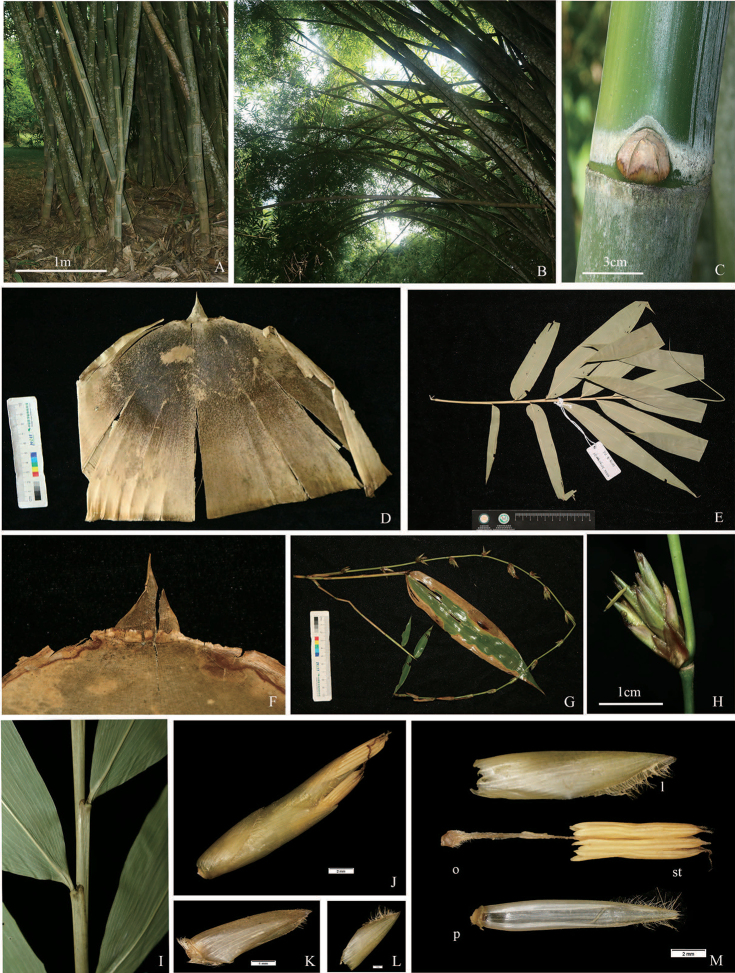
*Gigantochloa glabrata* N. H. Xia & Y. Zeng ex D. Z. Li & Z. C. Xu **A–C** culm **D, F** culm sheath **E, I** leaf **G** flower branches **H, J** pseudospikelet **K, L** glume **M** lemma(l), palea(p), ovary(o), stamens(st). Scale bars: 1 m (**A**); 3 cm (**C**); 1 cm (**E, H**).

“少毛巨竹”(Shao Mao Ju Zhu) Figures 1 , 2

Gigantochloa glabrata N. H. Xia & Y. Zeng in Y. Zeng Taxonomic Studies of Gigantochloa in China 36. 2014. *nom. nud.*. ‘Type’: CHINA. Yunnan: Xishuangbanna Tropical Botanical Garden (XTBG), Menglun, cultivated, 31 Aug 2012, *Y Zeng 17* (‘holotype’, IBSC).
= Gigantochloa albociliata auct. non (Munro) Kurz: C. J. Hsueh & J. L. Sun in Keng f. & Z. P. Wang, Fl. Reippubl. Poppularis. Sin. 9(1): 198. pl. 50, 1–11. 1996; D. Z. Li & Stapleton in Z. Y. Wu, P. H. Raven & D. Y. Hong, Fl. China 22: 47. 2006; L. B. Zhang in C. Y. Wu, P. H. Raven & D. Y. Hong, Fl. China Illustr. 22: 46. fig. 46:1–11, 2007.

#### Diagnosis.

*Gigantochloa glabrata* has erect culm sheath blade and the culm sheath covered with sparsely deciduous setae, with truncate apex. It is morphologically similar to *G. albociliata* and *G. levis*, but can be easily distinguished from them by having erect blades; culm sheath ligule 4–6 mm high, truncate, denticulate; a ring of white tomentum on the intranode and below the node (Table 1).

**Table 1.** Morphological differences between *Gigantochloa glabrata*, *G. albociliata* and *G. levis*.

Characters	*G. glabrata*	*G. albociliata*	*G. levis*
Diameter of culm	5–9 cm	1–5 cm	7–12 cm
Internode	yellow striped	white striped	not striped
Hairy ring	with a white hairy ring and below the node	without hairy ring	one brown hairy ring below the node
Culm sheath blade	erect	reflexed	reflexed
Culm sheath ligule	4–6 mm, truncate, denticulate	10–17 mm, convex in the middle, denticulate	9–14 mm, deep lacerations, bristle
Pseudospikelet	12–18 × 2–3 mm, lanceolate, straight	13–20 × 2–2.5 mm, slender, curved	11–12 × 3–4 mm, ovate, straight

#### Type.

China. Yunnan: Xishuangbanna Tropical Botanical Garden (XTBG), Menglun, Mengla, 101.2522°E, 21.9303°N, 514 m alt., introduction no. 00.1978.0594, 22 August 2019, *Xuzc2019041* (holotype, KUN!).

#### Description.

Sympodial bamboo, loosely tufted. Rhizomes pachymorph. Culms erect, lower nodes with verticillate aerial roots, apically pendulous, 9–14 m tall, 5–9 cm in diameter; internodes terete, greyish-green, yellow striped, 20–40 cm long, wall 7–12 mm thick, culm surface initially densely covered with white to brown hairs when young and glabrous or patchy smudge later; nodes inconspicuous, internode 7–10 mm tall, with a ring of white tomentum at the intranode and below the node. Culm sheaths deciduous, leathery, adaxially glabrous, abaxially sparsely hispidous with brown to black deciduous hairs, strigose, 20–28 cm long, hay colour, with truncate apex; auricles narrowly falcate, 7–10 mm wide, 1–2 mm tall; ligules 4–6 mm tall, denticulate; blades triangular, erect, 4–7 cm long, 1/2 as wide as the apex of culm sheaths. Bud ovate, branching high, from 3–4 m above ground, branches several, one dominant. Foliage leaves 8–12 per ultimate branchlet, usually 10; sheaths initially sparsely white hairy and later glabrous, keeled; auricles inconspicuous; ligules ca. 2 mm tall, entire or split; collar with external ligule; blades lanceolate, 10–28 (-40) cm × 2–4 cm, base cuneate, glabrous, margins serrulate, secondary veins 7–11 pairs, pseudopetioles 2–4 mm long.

Inflorescence iterauctant; flowering branches pendulous, leafless, with clusters of 4–8 (-20) large fertile pseudospikelets mixed with a few small sterile ones at each node, subtended by glumaceous bracts; internodes 2–10 cm long, covered with white deciduous hairs. Pseudospikelets narrowly ovate, light green, 12–18 mm long, 2–3 mm wide; fertile ones sessile, perfect fertile florets 2–4, with diminished florets at the apex; disarticulated above glumes, but not between florets; rhachilla internodes compressed between florets. Glumes 2–3, broadly ovate, persistent, veined, 5–9 mm long, 4–6 mm wide, margins ciliated at upper half. Fertile lemma lanceolate, 14–16 mm long, chartaceous, apex mucronate, glabrous abaxially, margins ciliated; palea oblanceolate, 2-keeled, equal length to lemma, keels and margins long ciliated; lodicules absent; anthers 6, 8–10 mm long, yellow, with a finely-toothed gradual apical tip 0.5–1 mm long, filaments united into a firm tube, 6–10 mm long; stigmas one, purple, plumose, ovary umbonate, pubescent apically. Caryopsis unknown.

#### Phenology.

New shoots May to August.

#### Distribution and habitat.

*Gigantochloa glabrata* is cultivated at the Bamboo Garden, XTBG, introduced from Mengyang Town, Jinghong City, Yunnan, China in 1978 with XTBG accession no. 00.1978.0594. However, we could not find it over a field survey in Mengyang area in 2019.

#### Etymology.

The specific epithet refers to the culm sheath covered with sparsely deciduous hairs.

#### Additional specimens examined.

China. Yunnan: Menghai Country, Daluo Town, Manka, 22 October 1978, *J. L. Sun 18070* (HITBC!); China. Yunnan: Mengla Country, Menglun Town, Bamboo Garden, XTBG, cultivated, 31 August 2012, *Y. Zeng 17* (IBSC!, with no flowering branches); ibid., 1 August 2007, *K. H. He* (何开红) *C130051* (HITBC!, HITBC0024167, flowering branches); ibid., 30 May 2020, *Xuzc2020001* (KUN!). All collections cited here (with the exception of *J. L. Sun 18070*) come from the same bamboo clump that was introduced to XTBG with the accession no. 00.1978.0594 in 1978 from Mengyang.

### Gigantochloa albociliata

Taxon classificationPlantaePoalesPoaceae

(Munro) Kurz, Prelim. Rep. Forest Pegu, App. A:136 1875 (‘albo-ciliata’)

CCB8387E-205F-556A-A8C4-F6177E70A81C

≡ Oxytenanthera albociliata Munro, Trans. Linn. Soc. London, 26: 129. 1868 (‘*albo-ciliata*’). –Type: Myanmar, Pegu, *Brandis 19* (syntype: K, K000710255!); Myanmar, Moulmein, *Falconer 27* (syntype: K, K000710256!).

#### Diagnosis.

*Gigantochloa albociliata* has reflexed culm sheath blades, culm sheath ligules 14–18 mm high, erose-toothed; culms have white hispid; dominant branches conspicuous.

#### Specimen examined.

China. Yunnan: Menghai Country, Daluo Town, 22 April 2016, *Liujx16024*, *Liujx16027* (KUN!); ibid., 10, December, 2016, *Liujx16056* (KUN!); ibid., Manka, 22 October 1978, *J. L. Sun 18069* (HITBC!); THAILAND. Sakon Nakhon, near Phu Pha National Park, 12 August 2018, *Liujx18009* (KUN!).

## Discussion

Xishuangbanna is a hotspot of biodiversity in the world and it is also the northern edge of the distribution of *Gigantochloa*. Our discovery not only increases the bamboo species diversity of this area, but also solves the problem of erroneous identifications and citations of *G. albociliata* in Chinese botanical literature for two decades, including the authoritative *Flora Reipublicae Popularis Sinicae* and *Flora of China*, as well as provincial and regional Floras.

## Supplementary Material

XML Treatment for Gigantochloa glabrata

XML Treatment for Gigantochloa albociliata
